# Gossip

**Published:** 1895-06

**Authors:** 


Secretary.

THE meetings of the Wyoming Valley Veterinary Medical Association, held during the past winter, have been fairly well attended. Tunckhannock, Scranton, and Wilkesbarre have all been favored with meetings, and a number of papers have been read, among the more important was one on "Traumatic Pericarditis," by Jacob Helmer, of Scranton, Pa.

THE present officers of the North Dakota Veterinary Medical Association are as follows: I. Turcot, *President;* B. C. Taylor, *Vice-President;* T. D. Hinebauch, *Secretary-Treasurer.*

ATTENTION is called (under "Society Proceedings") to a proposed association of the Alumni of the American Veterinary College in Pennsylvania. The Secretary *pro tem.*, Dr. Rhoads, earnestly requests as large an attendance as possible, and we thoroughly indorse the movement. It is in the right direction, and the American Veterinary College deserves well at the hands of her graduates.

MAINE veterinarians should give their energetic and earnest-working officers a stronger support, and thus place their Association in a more thorough working condition alongside of its neighbors in Massachusetts and New Hampshire.

DR. LEONARD PEARSON will be among those who will read papers at the coming meeting of the Keystone Veterinary Medical Association at Lansdowne. A generous invitation is extended the profession in the vicinity of Philadelphia to be present on the evening of June 11, 1895.

